# A Comparison of Machine Learning-Based Models and a Simple Clinical Bedside Tool to Predict Morbidity and Mortality After Gastrointestinal Cancer Surgery in the Elderly

**DOI:** 10.3390/bioengineering12050544

**Published:** 2025-05-19

**Authors:** Barbara Frezza, Mario Cesare Nurchis, Gabriella Teresa Capolupo, Filippo Carannante, Marco De Prizio, Fabio Rondelli, Danilo Alunni Fegatelli, Alessio Gili, Luca Lepre, Gianluca Costa

**Affiliations:** 1 General Surgery Unit, San Donato Hospital, Azienda USL Toscana Sud-Est, 52100 Arezzo, Italy; barbara.frezza@uslsudest.toscana.it (B.F.); marco.deprizio@uslsudest.toscana.it (M.D.P.); 2 Department of Life Sciences, Health and Health Professions, Link Campus University, 00165 Roma, Italy; m.nurchis@unilink.it (M.C.N.); d.alunnifegatelli@unilink.it (D.A.F.); a.gili@unilink.it (A.G.); 3 Operative Research Unit of Colorectal Surgery, Fondazione Policlinico Universitario Campus Bio-Medico, 00128 Roma, Italy; g.capolupo@policlinicocampus.it (G.T.C.); f.carannante@policlinicocampus.it (F.C.); 4 General Surgery and Surgical Specialties Unit, Santa Maria Hospital Terni, Teaching Hospital of Perugia University, 05100 Perugia, Italy; fabio.rondelli@unipg.it; 5 General and Emergency Surgery Unit, Santo Spirito in Sassia Hospital, ASL RM1, 00193 Roma, Italy; luca.lepre@aslroma1.it

**Keywords:** cancer, surgery, elderly, frailty, score, machine learning, postoperative outcome

## Abstract

Frailty in the elderly population is associated with increased vulnerability to stressors, including surgical interventions. This study compared machine learning (ML) models with a clinical bedside tool, the Gastrointestinal Surgery Frailty Index (GiS-FI), for predicting mortality and morbidity in elderly patients undergoing gastrointestinal cancer surgery. In a multicenter analysis of 937 patients aged ≥65 years, the performance of various predictive models including Random Forest (RF), Least Absolute Shrinkage and Selection Operator (LASSO), Stepwise Regression, K-Nearest Neighbors, Neural Network, and Support Vector Machine algorithms were evaluated. The overall 30-day mortality and morbidity rates were 6.1% and 35.7%, respectively. For mortality prediction, the RF model demonstrated superior performance with an AUC of 0.822 (95% CI 0.714–0.931), outperforming the GiS-FI score (AUC = 0.772, 95% CI 0.675–0.868). For morbidity prediction, all models showed more modest discrimination, with stepwise regression and LASSO regression achieving the highest performance (AUCs of 0.652 and 0.647, respectively). Our findings suggest that ML approaches, particularly RF algorithm, offer enhanced predictive accuracy compared to traditional clinical scores for mortality risk assessment in elderly cancer patients undergoing gastrointestinal surgery. These advanced analytical tools could provide valuable decision support for surgical risk stratification in this vulnerable population.

## 1. Introduction

In 2022, the European Union saw approximately 40 million surgical procedures performed annually, with a growing trend despite the pandemic-related disruptions [1]. Furthermore, as the global population ages, the proportion of individuals aged 65 years or older is projected to increase significantly. According to the United Nations, this demographic is expected to rise from 9.7% in 2022 to 16.4% by 2050, meaning that one in six people worldwide will be aged 65 or over by mid-century. Older adults facing urgent or emergency surgical procedures are at an increased risk of unfavorable outcomes due to a higher prevalence of comorbidities and elevated levels of frailty [2]. Frailty has emerged as a significant predictor of adverse surgical outcomes in the elderly. Defined as a multifactorial clinical syndrome characterized by diminished physiological reserves and increased vulnerability to stressors [3], frailty is associated with metabolic dysregulation, chronic inflammation, immunodeficiency, and hormonal imbalances [4].

Assessing the potential postoperative complications and survival risks for older patients undergoing time-sensitive surgical interventions is essential for developing informed, patient-centered care strategies in an increasingly aging healthcare landscape.

Recent studies have highlighted the predictive value of preoperative frailty assessments in determining length of hospital stay, operative risk, and other surgical outcomes in older patients [5,6,7,8].

Traditional risk assessment tools, such as the Emergency Surgery Frailty Index (EmSFI), have been developed to stratify surgical risk in elderly patients undergoing emergency procedures [9]. The EmSFI is a bedside tool designed to predict mortality and morbidity by evaluating frailty in this target population. While useful, these traditional models may not fully capture the complexity of frailty and its impact on surgical outcomes. Moreover, the implementation of these analytical models is constrained by their inherent complexity, which involves managing numerous interdependent variables and creating significant challenges in data collection and interpretation. In addition, these models predominantly rely on conventional methodological approaches, potentially overlooking the transformative capabilities offered by contemporary technological innovations and advanced data analysis techniques [10,11]. Machine learning (ML) represents a sophisticated analytical methodology that distinguishes itself from conventional approaches by its capacity for autonomous learning and continuous performance enhancement without manual programming interventions. Empirical evidence demonstrates that these advanced analytical models may outperform traditional statistical approaches in predictive accuracy and performance metrics, representing a significant methodological advancement in data-driven research and analysis [12,13,14]. Recently, ML algorithms have been employed to create electronic health record-based risk prediction models, such as the AGES score, which have shown excellent discrimination in forecasting major postoperative complications and mortality in geriatric surgical patients.

Given the increasing number of older adults requiring emergency surgical interventions, there is a pressing need to integrate novel statistical approaches, including ML techniques, into preoperative risk assessment. Through comprehensive analysis of extensive clinical datasets, these innovative techniques empower health professionals with advanced predictive capabilities, enabling more precise assessments of risk and potential progression trajectories.

In this study, we aim to compare the performance of ML models with traditional scoring systems (i.e., EmSFI) in predicting adverse outcomes in older adults undergoing emergency surgery.

## 2. Materials and Methods

### 2.1. Study Design and Setting

In previous studies, we developed a Frailty Index, called the Emergency Surgery Frailty Index (EmSFI) [9,15]. Briefly, nine variables have been identified, on which the EmSFI index is based. Each variable is associated with a score from 0 to 1, respectively, related to the absence or presence of the variable under examination, or from 0 to 2 depending on the clinical severity, according to the following conditions: 0 if absent, 1 if mild deficit or associated disease, and 2 if moderate/severe deficit or associated disease. The EmSFI is then calculated for each patient by adding up the scores obtained for each domain, being able to obtain a score ranging from 0 to 14 points. This statistical analysis allowed us to stratify patients in three classes according to the developed index: EmSFI < 3: low-risk class also considered not frail; EmSFI 4–7: moderate-risk class or frailty; and EmSFI: >8 high-risk class or severe frailty. Following the development of the EmSFI score, several centers validated and adopted it in their clinical emergency practice. In the present paper, we used our EmSFI as originally developed and then simply renamed it to the Gastrointestinal Surgery Frailty Index (GiS-FI), applied to digestive tract cancer surgery both in emergency and elective surgery settings, and compared it with several machine learning models.

### 2.2. Study Population and Data Sources

For the aim of the present study, as regards elective surgery, we reviewed the electronic health records of the General Surgery Unit at San Donato Hospital in Arezzo, of the General Surgery and Surgical Specialties Unit at Santa Maria Hospital in Terni, and of the Colorectal Clinical and Research Unit at Fondazione Policlinico Universitario Campus Bio-Medico, in Rome. With regard to emergency surgery, we reviewed the database of two previous studies [15,16]. We initially retrieved the records of all the patients aged >65 years with ICD-9-CM codes ranging from 150.x to 154.x or code 159.x undergoing surgery from October 2017 to October 2024. Furthermore, those who underwent procedures performed by junior surgeons or residents, patients submitted to palliative surgery, or those with pathological evidence of inadequate oncological surgery were discarded. Other exclusion criteria were the following: tumor different from adenocarcinoma; age < 65 years; previous abdominal surgery; patients already considered for the development and validation of the EmSFI score; and patients participating in other randomized or interventional clinical trials. Records with more than 5% of missing data or patients lacking data regarding frailty were also excluded. Missing data were handled using complete case analysis (CCA), whereby only observations with no missing values for the variables included in each model were retained. This method was applied under the assumption that data were missing completely at random (MCAR). Although the patient’s demographic information was collected, all data were anonymized before analysis by an IT specialist, even for center identification.

### 2.3. Patients’ Characteristics, Preoperative Variables, and Objectives of This Study

The data collected included patient demographic characteristics and clinical variables, procedure details, and outcomes. Demographic variables and clinical data included the following: age, gender, weight, height, body mass index (BMI), Glasgow Coma Scale (GCS), heart rate, systolic blood pressure, medical and surgical history (comorbidities), common preoperative biochemical blood examination, including C-Reactive Protein (CPR), and arterial blood gas analysis. All types of interventions performed with either an open or laparoscopic approach were considered, including laparoscopic interventions converted to open procedures. Comorbidity was recorded if the condition was being medically treated at the time of admission or if previous treatment for the condition was described in the admission report. Systemic inflammatory response syndrome (SIRS) was evaluated according to the original consensus study (Sepsis-1). SIRS criteria ≥ 2 met the definition of SIRS. The value concerning the item “solid tumor” was withdrawn from the calculation of all the scores if that was the indication for the index procedure. Arterial hypertension was not included in the models because, being present in half of the patients, it could be equivalent to chance. Postoperative complications were reported and categorized according to the Clavien–Dindo (C-D) classification system by the study leader in each of the participating centers [17].

Morbidity and mortality were considered as any “in-hospital” adverse event occurring in the postoperative period irrespective of the time elapsed from the index procedure. Concerning the objective of this study, the entire study cohort was first investigated to validate and compare our tool with the machine learning models regardless of the organ affected by cancer or of the procedure performed. The comparative analysis aimed to determine whether advanced statistical and machine learning approaches could enhance predictive accuracy relative to our previously established score. Furthermore, the population was divided into two subgroups based on the procedural setting (i.e., emergency and elective). Due to the design of the studies from which we retrieved the patients, there was no uniform standardized protocol either for the procedure performed or for the surgical approach adopted or for the perioperative therapy followed. However, because the procedures and approaches and enhanced recovery after surgery (ERAS) care were almost equally distributed through the facilities, the lack of uniformity was not considered a bias.

### 2.4. Statistical Analysis

Descriptive statistics were used to summarize the study data. Continuous variables were reported as mean and standard deviation or median and interquartile range, while categorical variables were presented as frequencies and proportions. To predict mortality and morbidity, multiple statistical and machine learning methods were employed, listed as follows: (i) *Least Absolute Shrinkage and Selection Operator* (LASSO) regression, which performs variable selection and regularization to prevent over-fitting [18]; (ii) *Stepwise Regression* (SW), an automated method for selecting predictive variables based on statistical significance [19]; (iii) *Random Forest* (RF), an ensemble learning technique that builds multiple decision trees to improve prediction accuracy [20]; (iv) *K-Nearest Neighbors* (KNN), a supervised learning algorithm that classifies new data points by identifying the most common category among the K closest samples in the feature space [21]; (v) a *Neural Network* (NN), a computational model inspired by the human brain, consisting of interconnected nodes (i.e., neurons) organized in layers that transform input data through weighted connections to produce output predictions [22]; (vi) and a *Support Vector Machine* (SVM), a classification algorithm that determines the optimal boundary between categories by finding the hyperplane that creates the widest possible gap between different classes [23].

For model development, we trained multiple ML algorithms using standard implementations in R. Hyperparameters were selected through internal cross-validation or based on default values commonly used in the literature. For LASSO logistic regression, we applied 10-fold cross-validation to determine the optimal regularization parameter, with the penalty term alpha set at 1. The RF model was trained using 500 trees and two variables considered at each split. Feature importance was assessed using the Gini index. The SVM model employed a radial basis function (RBF) kernel, with a grid search performed over cost values of 0.1, 1, 10, 100, and 1000 and gamma values of 0.5, 1, 2, 3, and 4; the best-performing parameter combination was selected and used to refit the model. For the KNN algorithm, we performed cross-validation over values of k equal to 3, 5, and 7, and we trained the final model using the optimal k. The NN was implemented with a single hidden layer of five neurons, a regularization parameter of 0.01, and a maximum of 500 iterations. Finally, we applied stepwise logistic regression to perform both forward and backward selection based on the Akaike Information Criterion (AIC), starting from a null model and considering the full model as the upper scope.

The dataset was divided into training and testing subsets using a 70/30 split ratio. This partitioning allowed models to be developed on the training dataset (i.e., 70% of available data) and independently validated on the testing dataset (i.e., 30% of available data), ensuring the unbiased evaluation of predictive performance and generalizability of the statistical and machine learning algorithms. We included all the features listed in Table 1 except for gender, GiSFI, arterial hypertension, and site. The performance of these predictive models was evaluated using the receiver operating characteristic (ROC) curves and their respective area under the curve (AUC) values. Model comparisons were conducted against the GiSFI score. For the interpretation of the values of the area below the ROC curve, the most common criteria reported were considered [24,25]. In general, an AUC of 0.5 suggests no discrimination, a value of 0.7 to 0.8 is deemed acceptable, 0.8 to 0.9 excellent, and more than 0.9 is deemed outstanding.

Calibration was evaluated by inspecting the intercept and slope of calibration models. The intercept reflects whether the model systematically over- or under-predicts risk (with an ideal value of 0), while the slope indicates the spread of predictions relative to actual outcomes (ideal value of 1, where >1 suggests underconfidence and <1 overconfidence).

For all statistical analyses, a *p*-value < 0.05 was considered statistically significant. Analyses were carried out by using R (R Core Team (2021). R: A language and environment for statistical computing. R Foundation for Statistical Computing, Vienna, Austria. URL https://www.R-project.org/ (accessed on 10 April 2025) version 4.4.3) with the “caret”, “nnet”, “class”, “randomForest”, and “glmnet” packages.

## 3. Results

### 3.1. Study Population

**Figure 1:**
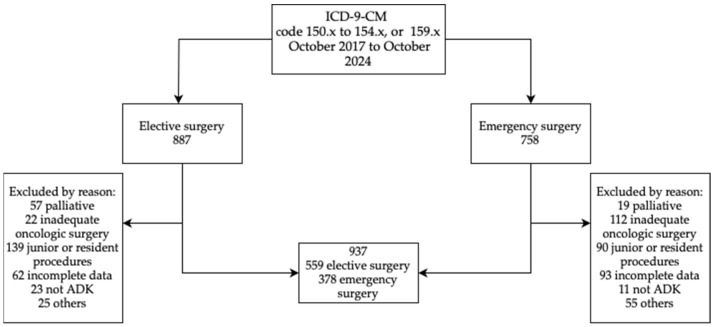
Flowchart of study population selection.

A total of 887 elective surgery patients and 758 emergency surgery patients were initially retrieved. A total of 937 patients, 559 (59.7%) elective and 378 (40.3%) emergency, finally fulfilled the inclusion criteria and were included in this study (Figure 1).

The mean age was 77.1 ± 6.9 years (IQR 72–82) and 78.0 ± 7.5 (IQR 72–84), with 52.4% and 51.1% of male patients in the elective and emergency groups, respectively.

The site of the tumor was in the large bowel in 600 (64.0%) patients, the stomach in 266 (28.4%), and other sites in 71 (7.6%) patients. Regarding the patients affected by neoplasms in other sites, we found tumors of the small bowel, of the distal esophagus (cardia), and of the duodenum in 47 (66.2%), 19 (26.8%), and 5 (7.0%) patients, respectively. The overall mortality and Clavien–Dindo I-IV morbidity rate was 6.1% (57 patients) and 35.7% (335 patients), respectively. Excluding minor complications (i.e., Clavien–Dindo I) the morbidity rate was 24.6% (231 patients). Serious complications (i.e., Clavien–Dindo ≥ III) occurred in 80 patients (8.5%).

Data about patient’s characteristics stratified by procedural setting are reported in Table 1.

### 3.2. Machine Learning Algorithms’ Performance

**Figure 2:**
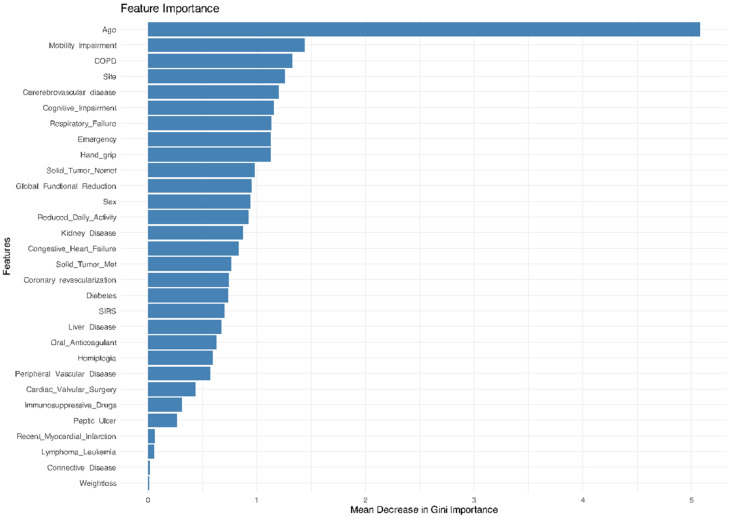
Variable importance plot based on the mean decrease in the Gini index from the RF model for mortality. Higher values reflect greater influence on node purity and model performance.

**Figure 3:**
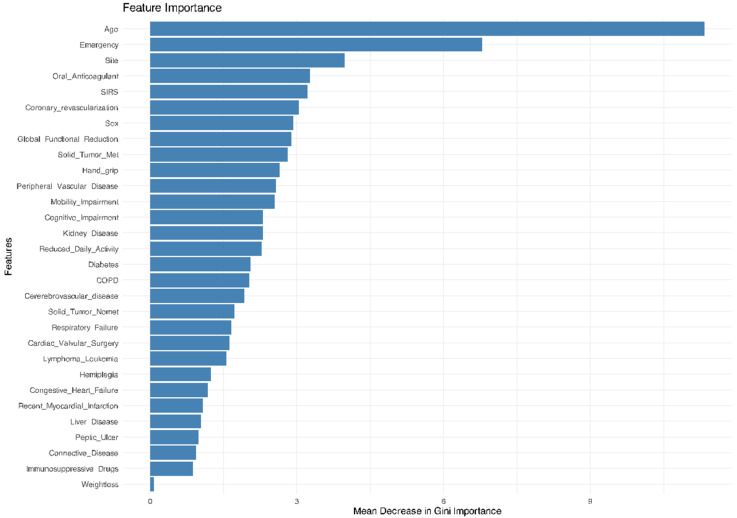
Variable importance plot based on the mean decrease in the Gini index from the RF model for morbidity. Higher values reflect greater influence on node purity and model performance.

**Figure 4:**
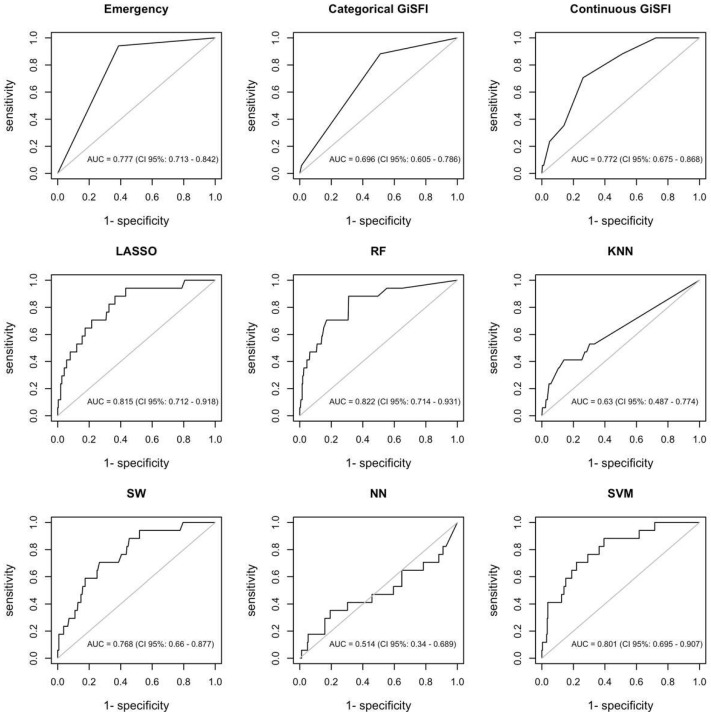
Evaluation of the predictive accuracy (AUROC) of different models for mortality in older adults undergoing oncologic gastrointestinal surgery. Abbreviations: GiSFI: Gastrointestinal Surgery Frailty Index; LASSO: Least Absolute Shrinkage and Selection Operator; RF: Random Forest; KNN: K-Nearest Neighbor; SW: Stepwise Regression; NN: Neural Network; SVM: Support Vector Machine.

**Figure 5:**
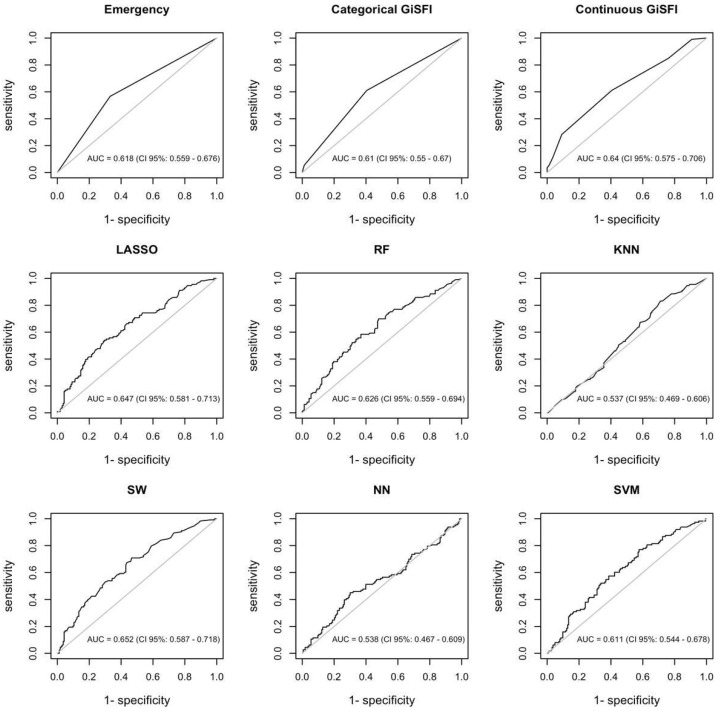
Evaluation of the predictive accuracy (AUROC) of different models for morbidity in older adults undergoing oncologic digestive tract surgery. Abbreviations: GiSFI: Gastrointestinal Surgery Frailty Index; LASSO: Least Absolute Shrinkage and Selection Operator; RF: Random Forest; KNN: K-Nearest Neighbor; SW: Stepwise Regression; NN: Neural Network; SVM: Support Vector Machine.

The GiSFI (ex EmSFI) score was employed as the traditional assessment tool while LASSO, RF, SW, KNN, NN, and SVM were adopted as ML model’s competitors. The RF model provides the best performance in predicting mortality, outperforming all the other models including the EmSFI score. Figure 2 and Figure 3 illustrate the relative importance of each variable in the RF model for both mortality and morbidity, ranked according to their contribution to predictive performance. Figure 4 and Figure 5 depict the performance of the different ML models in predicting mortality and morbidity in older adults undergoing emergency and elective surgery.

As illustrated in Figure 4, the AUC values for mortality ranges from 0.822 (95% CI 0.714–0.931) for the RF model to 0.514 (95% CI 0.340–0.689) for the NN model. The standard EmSFI score performed rather well (AUC = 0.772, 95% CI 0.675–0.868).

As concerns morbidity, prediction results are less accurate, as reflected by lower AUC values compared to those for mortality (Figure 5). Regression methods (i.e., SW and LASSO) outperform all the other algorithms. Particularly, AUC estimates amount to 0.652 (95% CI 0.587–0.718) and 0.647 (95% 0.581–0.713) for SW and LASSO, respectively.

In relation to model calibrations, overall, LASSO regression consistently shows the best calibration performance across both outcomes, outperforming traditional tools and other ML models. The GiSFI tends to overpredict risk and exhibit underconfident behavior, suggesting a need for recalibration or augmentation with more flexible models.

## 4. Discussion

Frailty is a prevalent and escalating concern among the aging population, characterized by diminished physiological reserves and increased vulnerability to stressors [26]. As global demographics shift, the healthcare system faces mounting challenges in managing frail older adults, particularly in acute care settings [27].

In the context of emergency surgery, frailty emerges as a critical determinant of patient outcomes. Studies have demonstrated that frailty, independent of chronological age, significantly correlates with increased postoperative mortality, morbidity, prolonged hospital stays, and loss of functional independence. For instance, the UK-based Emergency Laparotomy and Frailty (ELF) study revealed that frail patients undergoing emergency laparotomy had a 90-day mortality rate of 19.5%, with higher Clinical Frailty Scale (CFS) scores associated with increased mortality and complications [28,29]. Moreover, frailty has been linked to adverse outcomes across various emergency surgical procedures. A systematic review and meta-analysis encompassing multiple studies found that frail patients aged ≥ 65 undergoing emergency general surgery had nearly three times the odds of 30-day mortality compared to their non-frail counterparts [30]. These findings underscore the imperative need for accurate preoperative assessment tools that can effectively identify surgical outcomes in older frail patients. Despite the availability of traditional scoring systems, their predictive accuracy in frail populations remains suboptimal. This gap highlights the potential role of advanced predictive models, such as machine learning algorithms, in enhancing risk stratification and guiding clinical decision-making for frail elderly patients facing emergency surgical interventions.

This study compared the predictive performance of several ML models with the EmSFI in a cohort of older adults undergoing emergency gastrointestinal cancer surgery. Among the ML approaches tested, the RF algorithm showed superior accuracy in predicting mortality, with an AUC of 0.822, outperforming EmSFI (AUC = 0.772). In contrast, the prediction of morbidity proved more challenging across all models, with LASSO and stepwise regression offering modest discrimination (AUCs of 0.647 and 0.652, respectively). This finding broadly supports the work of a study in this area aimed at developing and validating the use of a machine learning approach to predict mortality following emergency general surgery. Gao et al. (2021) found that the ML algorithm better performed compared with the American Society of Anesthesiologists (ASA) classification, American College of Surgeon Surgical Risk Calculator (ACS-SRC), and modified frailty index (mFI) [31]. Furthermore, these results are consistent with those of Lee et al. [10], who conducted a cohort study in South Korea adopting extreme gradient boosting to predict 90-day postoperative mortality in patients over 75 years undergoing emergency surgery. This study highlighted that the ML model significantly outperformed traditional frailty assessment tools like the Operation Frailty Risk Score (OFRS) and Hospital Frailty Risk Score (HFRS) [10]. Although in different surgical scenarios, our findings agree with those obtained by Tang et al. [32] and Pean et al. [33]. The former research demonstrated the high efficiency of extreme gradient boosting machine and random forest models in predicting postoperative pneumonia in patients aged ≥ 80 undergoing hip fracture surgery [32]. The latter study showed that ML models outperformed traditional risk assessment indices (e.g., CARDE-B, 5-Item, and 6-Item mFI score) in predicting postoperative 30-day mortality after revision total hip and knee arthroplasty [33]. While the aforementioned analyses focus on individual primary studies, it is also valuable to consider broader evidence from secondary research. In this context, Wang et al. [34] conducted a comprehensive systematic review and meta-analysis comparing ML models to traditional algorithms in predicting postoperative outcomes for gastrointestinal surgery patients. Analyzing 38 studies encompassing 62 simple logistic regression models and 143 ML models, the study found that ML models had a significantly higher AUC compared to traditional models (ΔAUC = 0.07; 95% CI: 0.04–0.09; *p* < 0.001), implying that ML approaches offer a superior discriminatory ability [34].

Based on the study findings and comparative analysis with similar existing studies, some main operative implications emerge. Traditional clinical risk scores, such as the EmSFI, are developed through expert consensus and simple statistical analyses. These models are often based on a limited set of variables selected for their clinical relevance and ease of use. While they offer interpretability and have been validated in various settings, their simplicity may limit their predictive accuracy, especially in complex patient populations. In contrast, ML models can process vast amounts of high-dimensional data, capturing complex, non-linear relationships among variables that may be overlooked in traditional models. Therefore, in this era of personalized medicine, the integration of ML models into clinical practice offers the potential for more accurate and individualized risk assessments. This enables comprehensive counseling and collaborative decision-making between physicians, patients, and their families regarding surgical management strategies. Furthermore, beyond facilitating discussions about surgical risks, ML models could assist clinicians in identifying patients at elevated risk who might benefit from targeted preoperative and perioperative protocols, as well as optimize resource allocation, particularly in emergency surgical settings where timely decision-making is critical.

This study’s findings should be read in light of the main limitations and strengths. This study’s retrospective design might have introduced inherent biases, such as selection bias and information bias, which could have affected the internal validity of the findings. Notwithstanding, a comprehensive, real-world clinical dataset, reflecting routine practice in the emergency surgical management of older adults, was used. This enhances the ecological validity of the findings and ensures that the models are trained and tested on data representative of the target population and clinical setting. Another caveat is the exclusion of patients with more than 5% missing data or with lacking frailty information from the analyses. Nonetheless, the stringent exclusion criteria contributed to high data quality and internal consistency, which are critical for training and validating predictive models. Additionally, we acknowledge that more advanced or specialized ML techniques, including ensemble boosting methods (e.g., XGBoost or LightGBM), deep learning architectures, or hybrid modeling approaches, were not explored. These may offer incremental improvements in predictive accuracy and should be considered in future investigations. Nevertheless, the suite of ML algorithms applied, implemented via commonly used R packages, was deliberately selected to balance model performance with computational tractability and interpretability.

Although we employed robust resampling techniques and partitioned the dataset into training and testing subsets, the results remain subject to potential overfitting and optimism bias. As such, external validation using independent cohorts is essential to ascertain the generalizability and real-world applicability of the ML models developed in this study. Future research should explore dynamic modeling strategies that incorporate perioperative and postoperative variables (e.g., vital signs, lab trends, complications occurring early during hospitalization). Time series or longitudinal ML approaches, such as recurrent neural networks or survival forests, may provide more nuanced predictions of adverse outcomes, particularly for morbidity.

Further investigation is warranted to evaluate how ML models perform within actual clinical workflows and whether they improve outcomes compared to traditional scoring systems when used prospectively. Implementation of science approaches could be employed to assess feasibility, user engagement, and impact decision-making in the emergency surgical setting. Furthermore, as healthcare data privacy concerns grow and regulations become more stringent, future research should explore decentralized approaches to ML model development, such as federated learning. This paradigm enables training algorithms across multiple institutions without exchanging patient-level data, addressing privacy concerns while leveraging larger and more diverse datasets. Recent innovations in this field, such as dynamic barycenter bridging networks for federated transfer fault diagnosis [35] and balance recovery with collaborative adaptation approaches for federated diagnosis in inconsistent data groups [36], demonstrate promising methodologies that could be adapted to surgical risk prediction. These techniques are particularly relevant for addressing challenges related to data heterogeneity across different clinical centers and could potentially improve model generalizability while maintaining privacy compliance.

## 5. Conclusions

This study shows that ML models outperform traditional clinical scoring systems such as the EmSFI in predicting postoperative mortality and, to a lesser extent, morbidity in older adults undergoing gastrointestinal cancer surgery. These findings highlight the potential of data-driven approaches to refine risk stratification in complex, frail populations. However, while ML models offer superior accuracy and flexibility, they are not intended to replace clinical judgment. Rather, they should be viewed as decision support tools that complement the experience and contextual understanding of physicians. Their integration into clinical practice must be guided by transparency, interpretability, and validation in real-world settings. As the healthcare system evolves toward personalized and precision-based care, combining algorithmic insights with expert knowledge may offer the most effective path forward for managing high-risk surgical patients.

## Figures and Tables

**Table 1:** Demographic and clinical characteristics of the study population.

	All	Elective Surgery	Emergency Surgery
	937	559	378
Age			
years, mean (SD)	77.5 (7.1)	77.1 (6.9)	78.0 (7.5)
years, median (IQR)	78 (72–83)	77 (72–82)	78 (72–84)
Gender, male, N (%)	486 (51.9)	293 (52.4)	193 (51.1)
GiSFI, mean (SD)	3.76 (1.70)	3.59 (1.66)	4.03 (1.71)
GiSFI, median (IQR)	3 (3–5)	3 (2–5)	4 (3–5)
GiSFI 0–3, N (%)	474 (50.6)	297 (53.1)	177 (46.8)
GiSFI 4–7, N (%)	436 (46.5)	250 (44.7)	186 (49.2)
GiSFI ≥ 8, N (%)	27 (2.9)	12 (2.1)	15 (4.0)
SIRS	90 (9.6)	0 (0.0)	90 (23.8)
Recent myocardial infarction (>30 days)	20 (2.1)	12 (2.1)	8 (2.1)
Congestive heart failure	26 (2.8)	10 (1.8)	16 (4.2)
Coronary revascularization (PCI or CABG)	169 (18.0)	78 (14.0)	91 (24.1)
Cardiac valvular surgery	53 (5.7)	26 (4.7)	27 (7.1)
Arterial hypertension	535 (57.1)	306 (54.7)	229 (60.6)
Peripheral vascular diseases	94 (10.0)	40 (7.2)	54 (14.3)
Cerebrovascular diseases	52 (5.5)	18 (3.2)	34 (9.0)
Oral anticoagulant	124 (13.2)	56 (10.0)	68 (18.0)
COPD	128 (13.7)	65 (11.6)	63 (16.7)
Chronic respiratory failure with home oxygen delivery	37 (3.9)	10 (1.8)	27 (7.1)
Other non-metastatic solid tumors	98 (10.5)	7 (1.3)	91 (24.1)
Other solid metastatic tumors	128 (13.7)	31 (5.5)	97 (25.7)
Lymphoma/Leukemia	20 (2.1)	8 (1.4)	12 (3.2)
Liver disease	27 (2.9)	10 (1.8)	17 (4.5)
Kidney disease	58 (6.2)	15 (2.7)	43 (11.4)
Diabetes	199 (21.2)	125 (22.4)	74 (19.6)
Peptic ulcer	23 (2.5)	2 (0.4)	21 (5.6)
Connective disease	10 (1.1)	8 (1.4)	2 (0.5)
Immunosuppressive drugs	13 (1.4)	6 (1.1)	7 (1.9)
Hemiplegia or other post-stroke sequelae	26 (2.8)	4 (0.7)	22 (5.8)
Cognitive impairment	75 (8.0)	25 (4.5)	50 (13.2)
Weight loss	2 (0.2)	2 (0.4)	0 (0.0)
Reduced daily activity	180 (19.2)	66 (11.8)	114 (30.2)
Mobility impairment (gait speed reduced or wheelchair)	118 (12.6)	41 (7.3)	77 (20.4)
Impaired hand grip	154 (16.4)	9 (1.6)	145 (38.4)
Reduction in global functional status	152 (16.2)	62 (11.1)	90 (23.8)
Morbidity, N (%)	335 (35.7)	175 (31.3)	160 (42.3)
Clavien-Dindo I	104 (11.1)	58 (10.4)	46 (12.2)
Clavien-Dindo II	151 (16.1)	74 (13.2)	77 (20.4)
Clavien-Dindo III	57 (6.1)	29 (5.2)	28 (4.4)
Clavien-Dindo IV	23 (2.4)	14 (2.5)	9 (2.4)
Mortality, N (%)	57 (6.1)	13 (2.3)	44 (11.6)
Site			
Large bowel	600 (64.0)	313 (56.0)	287 (75.9)
Stomach	266 (28.4)	231 (41.3)	35 (9.3)
Other	71 (7.6)	15 (2.7)	56 (14.8)

Abbreviation: GiSFI: Gastrointestinal Surgery Frailty Index; PCI: percutaneous coronary interventions; CABG: coronary artery bypass graft surgery; SIRS: systemic inflammatory response syndrome; COPD: chronic obstructive pulmonary disease.

## Data Availability

The raw data supporting the conclusions of this article will be made available by the authors on request.
